# Betel nut chewing history is an independent prognosticator for smoking patients with locally advanced stage IV head and neck squamous cell carcinoma receiving induction chemotherapy with docetaxel, cisplatin, and fluorouracil

**DOI:** 10.1186/s12957-016-0844-2

**Published:** 2016-03-22

**Authors:** Yan-Ye Su, Chih-Yen Chien, Sheng-Dean Luo, Tai-Lin Huang, Wei-Che Lin, Fu-Min Fang, Tai-Jan Chiu, Yen-Hao Chen, Chi-Chih Lai, Cheng-Ming Hsu, Shau-Hsuan Li

**Affiliations:** Department of Otolaryngology, Kaohsiung Chang Gung Memorial Hospital and Chang Gung University College of Medicine, Kaohsiung, Taiwan; Department of Hematology-Oncology, Kaohsiung Chang Gung Memorial Hospital and Chang Gung University College of Medicine, No.123, Dapi Rd., Niaosong Dist. Kaohsiung City, 833 Taiwan (R.O.C.); Department of Diagnostic Radiology, Kaohsiung Chang Gung Memorial Hospital and Chang Gung University College of Medicine, Kaohsiung, Taiwan; Department of Radiation Oncology, Kaohsiung Chang Gung Memorial Hospital and Chang Gung University College of Medicine, Kaohsiung, Taiwan; Kaohsiung Chang Gung Head and Neck Oncology Group, Cancer Center, Kaohsiung Chang Gung Memorial Hospital, Kaohsiung, Taiwan

**Keywords:** Head and neck cancer, Squamous cell carcinoma, Betel nut, Smoking, Induction chemotherapy

## Abstract

**Background:**

Smoking and betel nut chewing are well-known risk factors for head and neck squamous cell carcinoma (HNSCC). Smoking is also a strong prognosticator for patients with locally advanced HNSCC receiving induction chemotherapy. Smoking with or without betel nut chewing is a common practice in Asia. However, little is known regarding whether betel nut chewing can serve as a prognostic factor for smoking patients with locally advanced HNSCC receiving induction chemotherapy. The aim of this study was to evaluate the prognostic impact of betel nut chewing in such patients receiving induction chemotherapy with docetaxel, cisplatin, and fluorouracil (TPF).

**Methods:**

From January 2010 to December 2012, we retrospectively analyzed 162 smoking patients with locally advanced HNSCC who received induction chemotherapy with TPF at our institution. Background characteristics, including a history of betel nut chewing, were analyzed as potential prognostic factors.

**Results:**

Among the 162 smoking patients, 131 patients (81 %) were betel nut chewers, while 31 (19 %) were non-betel nut chewers. One hundred fifty-six (96 %) were men, and 6 (4 %) were women. The median age was 53 years. The overall response rates to induction chemotherapy were 57 and 77 % in patients with and without betel nut chewing history, respectively (*P* = 0.038). The 2-year progression survival rates were 37 and 67 % in patients with and without betel nut chewing history, respectively (*P* = 0.004). The 2-year overall survival rates were 47 and 71 % in patients with and without betel nut chewing history, respectively (*P* = 0.017). Betel nut chewing history was independently associated with a poor response to induction chemotherapy, an inferior progression-free survival rate, and a poor overall survival rate.

**Conclusions:**

Our results indicate that betel nut chewing history is independently associated with poor prognosis in smoking patients with locally advanced HNSCC receiving induction chemotherapy with TPF. Further investigation is warranted to explain this effect of betel nut chewing history on these patients’ prognosis.

## Background

Head and neck squamous cell carcinoma (HNSCC) is one of the 10 leading cancers by incidence in the world [1]. Clinically, more than half of HNSCC patients present with locally advanced disease. Currently, induction chemotherapy is one of the treatment modalities for patients with locally advanced HNSCC [2–4]. A meta-analysis of chemotherapy in head and neck cancer revealed that when induction chemotherapy was included in the treatment of patients with locally advanced HNSCC, it was associated with an improvement in overall survival [3]. Recently, the randomized trial and meta-analysis [4–6] revealed that compared with the conventional regimen of cisplatin and fluorouracil (PF), the novel induction chemotherapy regimen of docetaxel, cisplatin, and fluorouracil (TPF) significantly increased survival for patients with locally advanced HNSCC.

Historically, tobacco smoking has been recognized as a major risk factor for HNSCC [7]. Previous studies [8–10] have reported that smoking was correlated with an inferior survival rate. Moreover, subjects with HNSCC who have a habit of smoking tobacco show a poor response to induction chemotherapy. While tobacco smoking and alcohol abuse are major risk factors of HNSCC over the world, betel nut chewing is an etiological factor that is highly specific to Asia and contributes to the increased incidence of HNSCC in the region [11]. Smoking, sometimes in addition to betel nut chewing is a prevalent habit in Asia [11–14]. However, little information is available on whether betel nut chewing serves as a prognostic factor for smokers who have contracted locally advanced HNSCC and are being treated with induction chemotherapy. The aim of this study is to evaluate the prognostic impact of betel nut chewing in smokers presenting with locally advanced HNSCC and being treated with induction chemotherapy and TPF.

## Methods

### Patient population

We retrospectively reviewed patients with locally advanced stage IV HNSCC who were treated with induction chemotherapy with TPF at Kaohsiung Chang Gung Memorial Hospital. The Institutional Review Board of Chang Gung Memorial Hospital approved the present study. The written consents were signed by the patients for their specimen and information to be stored in the hospital and used for research. The clinical staging was determined by a multidisciplinary team, including head and neck surgeons, medical oncologists, radiation oncologists, and radiologists according to the 7th American Joint Committee on Cancer (AJCC) staging system. Patients receiving induction chemotherapy with TPF were required to have Eastern Cooperative Oncology Group (ECOG) performance status 0 or 1, adequate bone marrow function (absolute neutrophil count ≥1.75 × 10^9^/L, platelet count ≥100 × 10^9^/L), hepatic function (serum total bilirubin ≤1.5 mg/dl and serum levels of aspartate aminotransferase and alanine aminotransferase ≤2.5 × ULN), and renal function (serum creatinine ≤1.5 mg/dl). Patients with synchronous cancers were excluded from this analysis. The medical history of these patients, including smoking, alcohol consumption, and betel nut chewing habits was obtained from their medical records, and patients who did not have a distinct history of smoking, alcohol consumption, or betel nut chewing were excluded from this study. Subjects who had consumed an alcoholic beverage ≧1 times per week for at least 6 months were considered to have a habit of alcohol consumption [15, 16]. Subjects who had smoked ≧10 tobacco cigarettes per week for at least 6 months were included as smokers [15, 16]. Subjects who had chewed ≧1 betel nut (measured as quid) per day for at least 6 months were included in the group of betel nut chewers [15, 16]. From January 2010 to December 2012, 194 patients with locally advanced stage IV head and neck squamous cell carcinoma were treated with induction chemotherapy with TPF in our center, and 16 patients were deleted from our analysis due to the lack of detailed information regarding smoking, alcohol, and betel nut chewing. Of the 178 patients with detailed information, 162 patients were smokers, whereas 16 patients were non-smokers. Background characteristics, including a history of betel nut chewing, were analyzed as potential prognostic factors in these 162 smokers.

### Treatment and assessment

Since the dosage of the TPF regimen used in those phase III clinical trials [4, 17] was in fact intolerable for most of the Asian patients with severe hematological toxicity [18], we modified the dosages of these agents to reduce their toxicity and increase tolerability. Docetaxel was administered at dosages of 60~65 mg/m^2^ by intravenous infusion for 1.5 h, cisplatin at dosages of 60~75 mg/m^2^ by intravenous infusion for 4 h, and 5-fluorouracil at dosages of 600~750 mg/m^2^ per 24 h as a 96-h continuous intravenous infusion, cycle that repeated every 3 weeks. All the patients received 2 to 3 cycles of induction chemotherapy with dose-modified TPF. Prophylactic granulocyte colony-stimulating factor and antibiotics were not routinely used; rather, their use was permitted in patients with febrile neutropenia. Within 3 to 6 weeks of completing the last cycle of induction chemotherapy, radiation was delivered over a 7-week period, using conventional fractionation (total dose: 66 to 70 Gy), which was concurrent with the weekly administration of 40 mg/m^2^ of cisplatin through intravenous infusion. Salvage surgery was performed 6 to 12 weeks after completion of chemoradiotherapy for patients who had residual disease after chemoradiotherapy and was also allowed for patients without response to induction chemotherapy with resectable disease. Tumor responses were assessed by clinical evaluation and imaging studies according to the Response Evaluation Criteria in Solid Tumors guideline version 1.0. Complete response (CR) was defined as disappearance of all target lesions. Partial response (PR) was defined as at least a 30 % decrease in the sum of the longest diameters of target lesions. Progressive disease (PD) was defined as at least a 20 % increase in the sum of the longest diameters of target lesions, taking as reference the smallest sum longest diameters recorded since the treatment started or the appearance of one or more new lesions. Stable disease (SD) was defined as neither sufficient shrinkage to qualify for PR nor sufficient increase to qualify for PD. The overall response rate was defined as the proportion of patients who achieve a CR or PR. Progression-free survival (PFS) was calculated from the date at which induction chemotherapy was administered for the first time until either the date of progression or death, whichever occurred earlier. The overall survival (OS) period was calculated from the date of diagnosis until death or the last follow-up.

### Immunohistochemistry

Immunohistochemistry staining was performed using an immunoperoxidase technique in patients whose pre-treatment biopsy specimens were available. The staining was performed on 4 μm slides, which were prepared from formalin-fixed, paraffin-embedded tissue sections with primary antibodies against the p16INK4 gene (BD Biosciences, San Jose, CA, USA). Briefly, after deparaffinization and rehydration, the antigen was retrieved by treating the slides with a solution of 10 mmol/L citrate buffer (pH 6.0); this process was performed in a hot water bath for 20 min at a temperature of 95 °C. The endogenous peroxidase activity was blocked for 15 min in 0.3 % hydrogen peroxide. After blocking the sections with 1 % goat serum for 1 h at room temperature, we incubated them overnight with primary antibodies for at least 18 h at 4 °C. Immunodetection was performed using the LSAB2 kit (Dako, Carpinteria, CA, USA). Thereafter, these sections were treated with 3-3′-diaminobenzidine dye and hematoxylin for color development and counterstaining, respectively. The staining assessment was independently carried out by two pathologists (S.L.W. and W.T.H.) who did not have any information about the clinicopathological features or prognoses of these subjects. A positive p16 expression in the tissue sections was defined as a strong nuclear staining in 50 % or more of the tumor cells [19].

### Statistical analysis

For patient data, statistical analysis was performed using the SPSS 17 software package. The chi-square test or Fisher’s exact test was used to compare data between the two groups. Multivariate analysis of the response of induction chemotherapy was performed by logistic regression, and all variables were entered into the model. For survival analysis, the Kaplan–Meier method was used for univariate analysis, and the difference between survival curves was tested by a log-rank test. All parameters were entered into Cox regression model to analyze their relative prognostic importance. For all analyses, two-sided tests of significance were used with *P* < 0.05 considered significant.

## Results

### Patient characteristics

A total of 162 smoking patients were collected in this study with a median age of 53 years (range, 31–72 years). The characteristics of 162 smoking patients with locally advanced stage IV HNSCC receiving induction chemotherapy with TPF are summarized in Table 1. Among them, 156 (96 %) were men and 6 (4 %) were women. Under T classification, 4 patients (3 %) were T1; 23 (14 %) were T2; 12 (7 %) were T3; 49 (30 %) were T4a; 70 (43 %) were T4b; and 4 (3 %) were Tx. Furthermore, under N classification, 28 patients (18 %) were N0; 20 (12 %) were N1; 7 (4 %) were N2a; 49 (30 %) were N2b; 41 (25 %) were N2c; and 17 (11 %) were N3. Additional analyses according to the AJCC 7th staging system demonstrated a stage IVA tumor for 80 (49 %) patients and stage IVB for 82 (51 %) patients. The primary tumor site was found in the hypopharynx in 19 (12 %) patients, larynx in 17 (10 %) patients, oropharynx in 60 (37 %) patients, oral cavity in 62 (38 %) patients, and unknown primary in 4 (3 %) patients. The p16 expression was negative in 128 (79 %) patients, positive in 3 (2 %) patients, and unknown status in 31 (19 %) patients. The overall response rate of these 162 patients was 61 %. Nine patients (6 %) achieved CR, 90 patients (55 %) achieved PR, 42 patients (26 %) had SD, and 21 patients (13 %) showed PD. Among the 162 smoking patients, 31 (19 %) were classified as non-betel nut chewers, while 131 patients (81 %) were betel nut chewers. The habit of betel nut chewing was not associated with any other clinicopathologic parameters, such as age, gender, primary tumor site, clinical 7th AJCC stage classification, clinical T classification, clinical N classification, and p16 expression (Table 2)

**Table 1:** Characteristics of 162 smoking patients with locally advanced stage IV head and neck squamous cell carcinoma receiving induction chemotherapy with TPF

Age		
	Median	53
	Mean	52.4
	Range	31–72
Sex		
	Male	156 (96 %)
	Female	6 (4 %)
Primary tumor site		
	Hypopharynx	19 (12 %)
	Larynx	17 (10 %)
	Oropharynx	60 (37 %)
	Oral cavity	62 (38 %)
	Unknown primary	4 (3 %)
Clinical T classification		
	T1	4 (3 %)
	T2	23 (14 %)
	T3	12 (7 %)
	T4a	49 (30 %)
	T4b	70 (43 %)
	Tx	4 (3 %)
Clinical N classification		
	N0	28 (18 %)
	N1	20 (12 %)
	N2a	7 (4 %)
	N2b	49 (30 %)
	N2c	41 (25 %)
	N3	17 (11 %)
Clinical 7th AJCC stage		
	IVA	80 (49 %)
	IVB	82 (51 %)
Alcohol		
	Absent	16 (10 %)
	Present	146 (90 %)
Betel nut chewing		
	Absent	31 (19 %)
	Present	131 (81 %)
p16 expression		
	Negative	128 (79 %)
	Positive	3 (2 %)
	Unknown	31 (19 %)
Response to induction chemotherapy		
	Complete response	9 (6 %)
	Partial response	90 (55 %)
	Stable disease	42 (26 %)
	Progression disease	21 (13 %)

*TPF* docetaxel, cisplatin, and fluorouracil

**Table 2:** Associations between betel nut chewing and clinicopathologic parameters in 162 smoking patients with locally advanced stage IV head and neck squamous cell carcinoma receiving induction chemotherapy with TPF

Parameters		Betel nut chewing		
		Absent	Present	*P* value
Age	<53 years old	13	68	0.32
	≧53 years old	18	63	
Sex	Male	29	127	0.32
	Female	2	4	
Clinical 7th AJCC stage	IVA	17	63	0.50
	IVB	14	68	
Clinical T classification	T1~T4a	19	73	0.57
	T4b	12	58	
Clinical N classification	N0/1	8	40	0.60
	N2/3	23	91	
Primary tumor site	Oral cavity	11	51	0.72
	Others	20	80	
Primary tumor site	Oropharynx	10	50	0.54
	Others	21	81	
Primary tumor site	Larynx/hypopharynx	9	27	0.31
	Others	22	104	
p16 expression	Negative	27	101	0.21
	Positive	1	2	
	Unknown	3	28	
Alcohol	Absent	6	10	0.086
	Present	25	121	

*TPF*, docetaxel, cisplatin, and fluorouracil

*х* ^2^ test, Fisher’s exact test, or *t* test was used for statistically analyzed

At the time of the last analysis, the median period of follow-up was 31 months (range, 24.1–55.3 months) for the 72 survivors. The median PFS and OS were 15.4 and 24.1 months, respectively. The 2-year PFS and OS were 43 and 51 %, respectively.

### Association between clinicopathological parameters and betel nut chewing history with the response to induction chemotherapy

Table 3 summarizes the relationship between clinicopathological parameters and their response to induction chemotherapy in 162 smoking patients receiving induction chemotherapy with TPF. Patients with a history of betel nut chewing were significantly (*P* = 0.038) associated with a poor response to induction chemotherapy. Compared with the 57 % response rate in patients with a history of betel nut chewing, the overall response (CR plus PR) rate to induction chemotherapy was 77 % in patients who did not have a history of betel nut chewing. The clinical N classification, N2/3, was also significantly (*P* = 0.045) correlated with a poor response to induction chemotherapy. The logistic model showed that the history of betel nut chewing (*P* = 0.04, odds ratio 2.742, 95 % confidence interval 1.050–7.166) and clinical N classification, N2/3 (*P* = 0.024, odds ratio 2.494, 95 % confidence interval 1.126–5.522), were independently correlated with the poor response to induction chemotherapy.

**Table 3:** Associations between the response of induction chemotherapy and clinicopathologic parameters in 162 smoking patients with locally advanced stage IV head and neck squamous cell carcinoma receiving induction chemotherapy with TPF

Parameters		Response to induction chemotherapy		
		CR/PR	SD/PD	*P* value
Age	<53 years old	47	34	0.42
	≧53 years old	52	29	
Sex	Male	96	60	0.68
	Female	3	3	
Clinical 7th AJCC stage	IVA	47	33	0.54
	IVB	52	30	
Clinical T classification	T1~T4a	55	37	0.69
	T4b	44	26	
Clinical N classification	N0/1	35	13	0.045*
	N2/3	64	50	
Primary tumor site	Oral cavity	35	27	0.34
	Others	64	36	
Primary tumor site	Oropharynx	40	20	0.27
	Others	59	43	
Primary tumor site	Larynx/hypopharynx	23	13	0.70
	Others	76	50	
Alcohol	Absent	10	6	0.90
	Present	89	57	
Betel nut chewing	Absent	24	7	0.038*
	Present	75	56	

*TPF* docetaxel, cisplatin, and fluorouracil; *CR* complete response; *PR* partial response; *SD* stable disease; *PD* progression disease

*Statistically significant. *х* ^2^ test, Fisher’s exact test, or *t* test was used for statistically analyzed

### Survival analyses

**Fig. 1:**
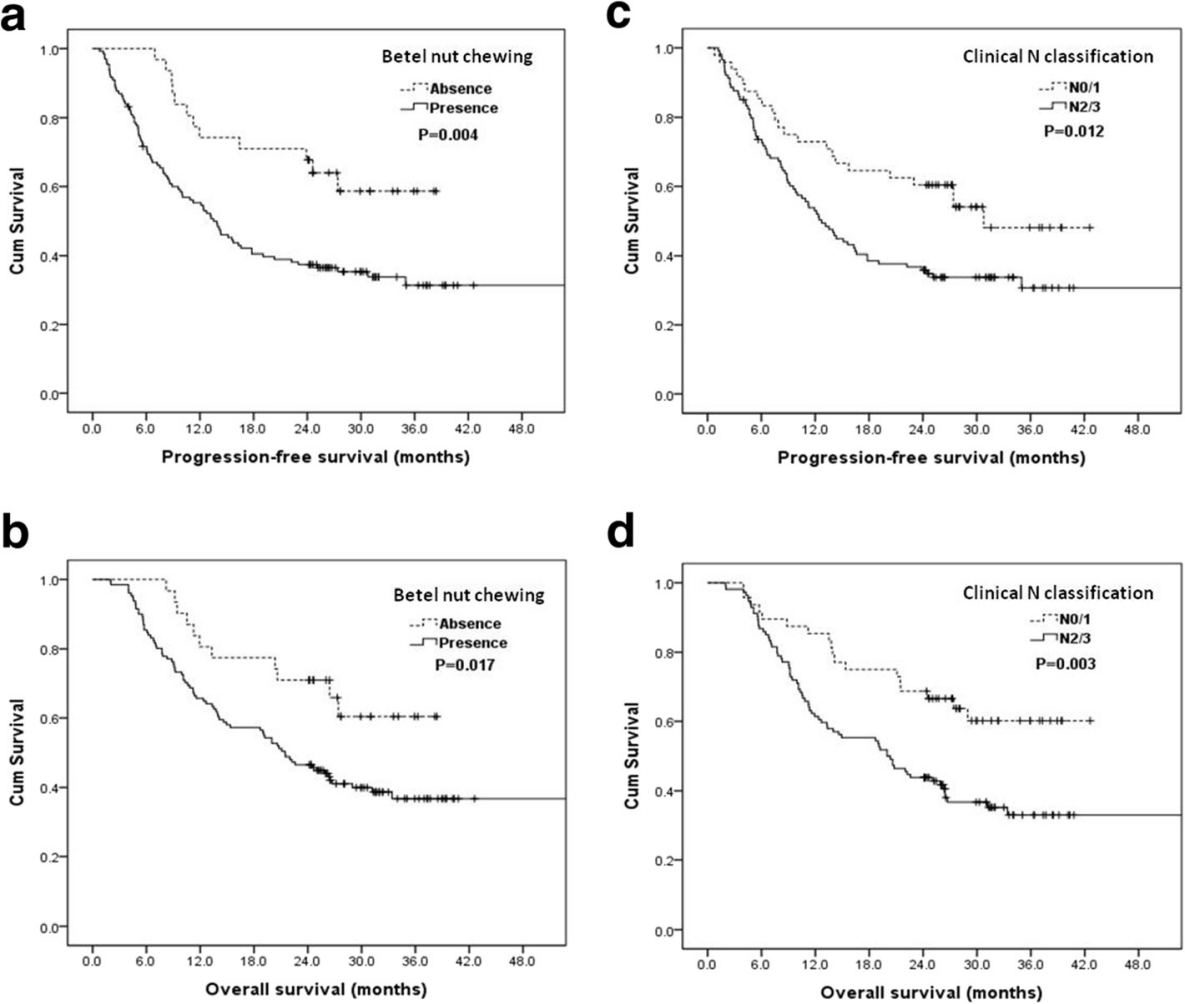
**a** Progression-free survival according to betel nut chewing status. **b** Progression-free survival according to clinical N classification. **c** Overall survival according to betel nut chewing status. **d** Overall survival according to clinical N classification

Table 4 displays the correlations between patient survival and various clinicopathological factors at the univariate level. Univariately, patients with a history of betel nut chewing (*P* = 0.004, Fig. 1a) and the clinical N classification, N2/3 (*P* = 0.012, Fig. 1b), were significantly associated with the worse PFS. Besides this, patients with a history of betel nut chewing (*P* = 0.017, Fig. 1c) and the clinical N classification, N2/3 (*P* = 0.003, Fig. 1d), were also significantly related to worse OS. The 2-year PFS and OS rates were 67 and 71 %, respectively, in patients who did not have a history of betel nut chewing, while the rates were 37 and 47 %, respectively, in patients with a history of betel nut chewing.

**Table 4:** Results of univariate log-rank analysis of prognostic factors for progression-free survival and overall survival in 162 smoking patients with locally advanced stage IV head and neck squamous cell carcinoma receiving induction chemotherapy with TPF

Factors	No. of patients	Progression-free survival (PFS)		Overall survival (OS)	
		2-year PFS rate (%)	*P* value	2-year OS rate (%)	*P* value
Age					
<53 years old	81	40	0.13	47	0.30
≧53 years old	81	46		56	
Clinical 7th AJCC stage					
IVA	80	46	0.62	58	0.30
IVB	82	41		45	
Clinical T classification					
T1 ~ 4a	92	44	0.77	55	0.49
T4b	70	42		46	
Clinical N classification					
N0/1	48	60	0.012*	69	0.003*
N2/3	114	36		44	
Primary tumor site					
Oral cavity	62	39	0.19	44	0.18
Others	100	46		56	
Primary tumor site					
Oropharynx	60	45	0.65	55	0.42
Others	102	42		49	
Primary tumor site					
Hypopharynx/Larynx	36	50	0.28	61	0.36
Others	126	41		48	
Alcohol					
Absent	16	56	0.25	69	0.29
Present	146	42		49	
Betel nut chewing					
Absent	31	67	0.004*	71	0.017*
Present	131	37		47	

*TPF* docetaxel, cisplatin, and fluorouracil

*Statistically significant

In a multivariate comparison, a history of betel nut chewing (*P* = 0.009, odds ratio 2.292, 95 % confidence interval 1.235–4.254) and clinical N classification of N2/3 (*P* = 0.003, odds ratio 2.145, 95 % confidence interval 1.285–3.579) represented an independent adverse prognosticator for the PFS rate. Moreover, a history of betel nut chewing (*P* = 0.031, odds ratio 2.033, 95 % confidence interval 1.066–3.879) and a clinical N classification of N2/3 (*P* < 0.001, odds ratio 2.760, 95 % confidence interval 1.582–4.816) were also an independent adverse prognosticators for the OS rate.

## Discussion

Tobacco smoking is an established risk factor of HNSCC all over the world [7]. In Taiwan, many people also have the habit of chewing betel nuts, which is the seed of the Areca palm (*Areca catechu*). It is estimated that nearly 2.8 million people chew betel nuts in Taiwan, and more men chew these nuts than women (9.8 vs. 1.6 %). The lifetime prevalence of this habit is as high as 15 % [20]. Around the world, 600 million people chew betel nuts, especially in India and Southeast Asia [21]. Several studies have shown that betel nut chewing is also a risk factor for developing HNSCC [11]. However, scarce evidence is available on whether these risk factors interact with major treatment modalities in HNSCC. Fountzilas et al. [8] reported that absence of smoking history was an important prognostic factor for complete response in 154 patients with locally advanced HNSCC receiving platinum-based induction chemotherapy. Kumar et al. [10] demonstrated that a history of smoking was associated with a poor response to induction chemotherapy and a poor survival rate in patients with oropharyngeal cancer. In Asia, many people have a smoking habit and some chew betel nuts as well [11–14]. However, no study has been carried out on a large scale to evaluate whether the history of betel nut chewing serves as a prognostic factor in smoking patients receiving induction chemotherapy for locally advanced HNSCC. This is why we conducted this study. We found significantly a poor response to induction chemotherapy in smoking patients with a history of betel nut chewing, compared to smoking patients without a history of betel nut chewing. Moreover, smoking patients with a history of betel nut chewing also had a significantly worse survival rate. To the best of our knowledge, this is the first evidence of this phenomenon in patients with locally advanced HNSCC receiving induction chemotherapy. Our result indicates that betel nut chewing might substantially affect a patient’s response to induction chemotherapy, leading to a poor treatment outcome.

Recently, induction chemotherapy has been commonly used in clinical practice for patients with locally advanced HNSCC. After induction chemotherapy, tumor response is observed in 60~75 % of patients with locally advanced HNSCC [4, 22]. Previous studies [22–24] reported that patients whose tumor did not respond to induction chemotherapy had an inferior survival compared with those whose tumor responded well to induction chemotherapy, indicating that the use of induction chemotherapy may benefit only a subgroup of patients. In addition, failed attempts to produce a response from induction chemotherapy may give rise to additional side effects. It may also postpone other therapeutic options, such as concurrent chemoradiotherapy or surgery. In the present study, a poor response to induction chemotherapy was seen in nearly half of smoking patients with a history of betel nut chewing. Further study is necessary to validate whether smoking patients with locally advanced HNSCC with a history of betel nut chewing could benefit from induction chemotherapy.

The reason why a history of betel nut chewing is associated with poor response to induction chemotherapy remains largely unknown. Overexpression of DNA repair enzymes due to betel nut chewing might be one possibility [25, 26]. O^6^-methylguanine-DNA methyltransferase (MGMT) is a DNA repair enzyme, which has been recognized as a therapeutic determinant of the efficacy of O^6^-alkylguanine alkylating drugs, including cisplatin [27]. Arecoline, a major betel nut alkaloid, is considered to be the most important substance in betel nuts, and preclinical study showed that arecoline can stimulate MGMT expression in primary human oral keratinocytes [25, 26]. The enzyme excision repair cross-complementation group 1 (ERCC1) plays an important role in the nucleotide excision repair pathway, and its expression has been associated with chemotherapy resistance [28]. Chiu et al. [28] reported that patients with a history of betel nut chewing had higher expression of ERCC1. These studies may explain the poor response to induction chemotherapy in patients with a history of betel nut chewing in the present study. Further studies are necessary to investigate the association between betel nut chewing, DNA repair enzyme, and the response to induction chemotherapy.

Our study has several limitations. First, the present study was a retrospective analysis. Substance use histories including smoking, alcohol, and betel nut chewing were obtained from medical record. We did not have questionnaires for these patients to gather further data. Besides, the reliability of patient’s history on substance use habits is also a limitation of our study. Furthermore, our observations were based on a relatively small number of patients.

## Conclusions

Smoking patients with locally advanced stage IV HNSCC with a history of betel nut chewing has a poor response to induction chemotherapy and worse prognosis. Further studies are necessary to validate our results and evaluate the underlying mechanism of different sensitivities to chemotherapy between patients with and without a history of betel nut chewing.

## Abbreviations

AJCC: American Joint Committee on Cancer
CR: complete response
ECOG: Eastern Cooperative Oncology Group
ERCC1: excision repair cross-complementation group 1
HNSCC: head and neck squamous cell carcinoma
MGMT: O^6^-methylguanine-DNA methyltransferase
OS: overall survival
PD: progression disease
PF: cisplatin and fluorouracil
PFS: progression-free survival
PR: partial response
SD: stable disease
TPF: docetaxel, cisplatin, and fluorouracil

## References

[1] Jemal A, Bray F, Center MM, Ferlay J, Ward E, Forman D (2011). Global cancer statistics. CA Cancer J Clin.

[2] Haddad RI, Shin DM (2008). Recent advances in head and neck cancer. N Engl J Med.

[3] Pignon JP, le Maitre A, Maillard E, Bourhis J (2009). Meta-analysis of chemotherapy in head and neck cancer (MACH-NC): an update on 93 randomised trials and 17,346 patients. Radiother Oncol.

[4] Vermorken JB, Remenar E, van Herpen C, Gorlia T, Mesia R, Degardin M, Stewart JS, Jelic S, Betka J, Preiss JH (2007). Cisplatin, fluorouracil, and docetaxel in unresectable head and neck cancer. N Engl J Med.

[5] Qin H, Luo J, Zhu YP, Xie HL, Yang WQ, Lei WB (2012). Combination of taxanes, cisplatin and fluorouracil as induction chemotherapy for locally advanced head and neck cancer: a meta-analysis. PLoS One.

[6] Lorch JH, Goloubeva O, Haddad RI, Cullen K, Sarlis N, Tishler R, Tan M, Fasciano J, Sammartino DE, Posner MR, Group TAXS (2011). Induction chemotherapy with cisplatin and fluorouracil alone or in combination with docetaxel in locally advanced squamous-cell cancer of the head and neck: long-term results of the TAX 324 randomised phase 3 trial. Lancet Oncol.

[7] D’Cruz A, Lin T, Anand AK, Atmakusuma D, Calaguas MJ, Chitapanarux I, Cho BC, Goh BC, Guo Y, Hsieh WS (2013). Consensus recommendations for management of head and neck cancer in Asian countries: a review of international guidelines. Oral Oncol.

[8] Fountzilas G, Kosmidis P, Avramidis V, Nikolaou A, Kalogera-Fountzila A, Makrantonakis P, Bacoyiannis C, Samantas E, Skarlos D, Daniilidis J (1997). Long-term survival data and prognostic factors of a complete response to chemotherapy in patients with head and neck cancer treated with platinum-based induction chemotherapy: a Hellenic Co-operative oncology Group study. Med Pediatr Oncol.

[9] Kawakita D, Hosono S, Ito H, Oze I, Watanabe M, Hanai N, Hasegawa Y, Tajima K, Murakami S, Tanaka H, Matsuo K (2012). Impact of smoking status on clinical outcome in oral cavity cancer patients. Oral Oncol.

[10] Kumar B, Cordell KG, Lee JS, Prince ME, Tran HH, Wolf GT, Urba SG, Worden FP, Chepeha DB, Teknos TN (2007). Response to therapy and outcomes in oropharyngeal cancer are associated with biomarkers including human papillomavirus, epidermal growth factor receptor, gender, and smoking. Int J Radiat Oncol Biol Phys.

[11] Park S, Bae J, Nam BH, Yoo KY (2008). Aetiology of cancer in Asia. Asian Pac J Cancer Prev.

[12] Chen PT, Kuan FC, Huang CE, Chen MF, Huang SH, Chen MC, Lee KD (2011). Incidence and patterns of second primary malignancies following oral cavity cancers in a prevalent area of betel-nut chewing: a population-based cohort of 26,166 patients in Taiwan. Jpn J Clin Oncol.

[13] Lin YS, Chu NF, Wu DM, Shen MH (2004). Prevalence and factors associated with the consumption of betel-nut among military conscripts in Taiwan. Eur J Epidemiol.

[14] Nagpal R, Nagpal N, Mehendiratta M, Marya CM, Rekhi A (2014). Usage of betel quid, areca nut, tobacco, alcohol and level of awareness towards their adverse effects on health in a north Indian rural population. Oral Health Dent Manag.

[15] Lin MY, Chen MC, Wu IC, Wu DC, Cheng YJ, Wu CC, Chai CY, Lee JM, Wu MT (2011). Areca users in combination with tobacco and alcohol use are associated with younger age of diagnosed esophageal cancer in Taiwanese men. PLoS ONE.

[16] Wu IC, Wu CC, Lu CY, Hsu WH, Wu MC, Lee JY, Chou SH, Lee JM, Chou YP, Wu DC, Wu MT (2013). Substance use (alcohol, areca nut and cigarette) is associated with poor prognosis of esophageal squamous cell carcinoma. PLoS ONE.

[17] Posner MR, Hershock DM, Blajman CR, Mickiewicz E, Winquist E, Gorbounova V, Tjulandin S, Shin DM, Cullen K, Ervin TJ (2007). Cisplatin and fluorouracil alone or with docetaxel in head and neck cancer. N Engl J Med.

[18] Ahn JS, Cho SH, Kim OK, Lee JK, Yang DH, Kim YK, Lee JJ, Lim SC, Kim HJ, Chung WK, Chung IJ (2007). The efficacy of an induction chemotherapy combination with docetaxel, cisplatin, and 5-FU followed by concurrent chemoradiotherapy in advanced head and neck cancer. Cancer Res Treat.

[19] Kuo KT, Hsiao CH, Lin CH, Kuo LT, Huang SH, Lin MC (2008). The biomarkers of human papillomavirus infection in tonsillar squamous cell carcinoma-molecular basis and predicting favorable outcome. Mod Pathol.

[20] Ko YC, Chiang TA, Chang SJ, Hsieh SF (1992). Prevalence of betel quid chewing habit in Taiwan and related sociodemographic factors. J Oral Pathol Med.

[21] Secretan B, Straif K, Baan R, Grosse Y, El Ghissassi F, Bouvard V, Benbrahim-Tallaa L, Guha N, Freeman C, Galichet L (2009). A review of human carcinogens—part E: tobacco, areca nut, alcohol, coal smoke, and salted fish. Lancet Oncol.

[22] Zhong LP, Zhang CP, Ren GX, Guo W, William WN, Sun J, Zhu HG, Tu WY, Li J, Cai YL (2013). Randomized phase III trial of induction chemotherapy with docetaxel, cisplatin, and fluorouracil followed by surgery versus up-front surgery in locally advanced resectable oral squamous cell carcinoma. J Clin Oncol.

[23] Vokes EE, Athanasiadis I (1996). Chemotherapy of squamous cell carcinoma of head and neck: the future is now. Ann Oncol.

[24] Kies MS, Boatright DH, Li G, Blumenschein G, El-Naggar AK, Brandon Gunn G, Lewin JS, Steinhaus GD, Sturgis EM (2012). Phase II trial of induction chemotherapy followed by surgery for squamous cell carcinoma of the oral tongue in young adults. Head Neck.

[25] Lee SS, Tsai CH, Yu CC, Ho YC, Hsu HI, Chang YC (2013). The expression of O(6)-methylguanine-DNA methyltransferase in human oral keratinocytes stimulated with arecoline. J Oral Pathol Med.

[26] Lu SY, Chang KW, Liu CJ, Tseng YH, Lu HH, Lee SY, Lin SC (2006). Ripe areca nut extract induces G1 phase arrests and senescence-associated phenotypes in normal human oral keratinocyte. Carcinogenesis.

[27] Maki Y, Murakami J, Asaumi J, Tsujigiwa H, Nagatsuka H, Kokeguchi S, Fukui K, Kawai N, Yanagi Y, Kuroda M (2005). Role of O6-methylguanine-DNA methyltransferase and effect of O6-benzylguanine on the anti-tumor activity of cis-diaminedichloroplatinum(II) in oral cancer cell lines. Oral Oncol.

[28] Chiu TJ, Chen CH, Chien CY, Li SH, Tsai HT, Chen YJ (2011). High ERCC1 expression predicts cisplatin-based chemotherapy resistance and poor outcome in unresectable squamous cell carcinoma of head and neck in a betel-chewing area. J Transl Med.

